# Effects of marine biofertilisation on Celtic bean carbon, nitrogen and sulphur isotopes: Implications for reconstructing past diet and farming practices

**DOI:** 10.1002/rcm.8985

**Published:** 2021-01-14

**Authors:** Darren R. Gröcke, Edward R. Treasure, Jonathan J. Lester, Kurt J. Gron, Mike J. Church

**Affiliations:** ^1^ Department of Earth Sciences Durham University South Road Durham DH1 3LE UK; ^2^ Department of Archaeology Durham University South Road Durham DH1 3LE UK

## Abstract

**Rationale:**

The application of fertilisers to crops can be monitored and assessed using stable isotope ratios. However, the application of marine biofertilisers (e.g., fish, macroalgae/seaweed) on crop stable isotope ratios has been rarely studied, despite widespread archaeological and historical evidence for the use of marine resources as a soil amendment.

**Methods:**

A heritage variety of Celtic bean, similar in size and shape to archaeobotanical macrofossils of *Vicia faba* L., was grown in three 1 × 0.5 m outdoor plots under three soil conditions: natural soil (control); natural soil mixed with macroalgae (seaweed); and 15 cm of natural soil placed on a layer of fish carcasses (Atlantic cod). These experiments were performed over two growing seasons in the same plots. At the end of each growing season, the plants were sampled, measured and analysed for carbon, nitrogen and sulphur stable isotope ratios (δ^13^C, δ^15^N, δ^34^S).

**Results:**

The bean plants freely uptake the newly bioavailable nutrients (nitrogen and sulphur) and incorporate a marine isotopic ratio into all tissues. The bean δ^15^N values ranged between 0.8‰ and 1.0‰ in the control experiment compared with 2‰ to 3‰ in the macroalgae crop and 8‰ to 17‰ in the cod fish experiment. Their δ^34^S values ranged between 5‰ and 7‰ in the control compared with 15‰ to 16‰ in the macroalgae crop and 9‰ to 12‰ in the cod fish crop. The beans became more ^13^C‐depleted (δ^13^C values: 1–1.5‰ lower) due to crop management practices.

**Conclusions:**

Humans and animals consuming plants grown with marine biofertilisers will incorporate a marine signature. Isotopic enrichment in nitrogen and sulphur using marine resources has significant implications when reconstructing diets and farming practices in archaeological populations.

## 1 INTRODUCTION

Evidence for deliberate soil amendment strategies, or the use of crop fertilisers, has been identified amongst the earliest farming communities in many areas of the world.^1^, ^2^ However, the continued use of synthetic and chemical fertilisers in modern environments is under increasing scrutiny due to climate and human‐induced changes in the Earth System.^3^ Consequently, greater emphasis is now being placed on the use of organic ‘traditional’ fertilisers such as animal manure or marine resources such as seaweed or fish.^4^ The effect of different fertilisers can be traced in archaeobotanical remains of crops using stable isotope ratios, although current research has predominantly focused on the impact of animal manure,^5^ despite evidence for the use of other biofertilisers.^6^ Hereafter, we use the term “biofertilisation” to categorise the use of natural biological materials for fertilisation compared with processed, synthetic or chemical fertilisers. Animal manure is likely to have been the most widely used form of soil amendment in both prehistoric and historic periods.^7^ However, a wide range of biofertilisers were potentially available including domestic refuse, human faeces or ‘nightsoil’, ashes, turves, animal products (bone, blood, hooves) and, of particular relevance here, marine resources such as shell sand, macroalgae (i.e., seaweed) and fish.^6^, ^7^

There is widespread archaeological and historical evidence for the use of seaweed as a fertiliser, especially throughout the medieval period in northern Europe along the Atlantic coast.^7^, ^8^, ^9^, ^10^, ^11^, ^12^ Eighteenth and nineteenth century farmers living near the English coast would apply seaweed (either in a fresh‐state or burnt/ashed) and fish to their crops, often mixed with household waste.^13^, ^14^, ^15^ In comparison, direct evidence for the use of fish (e.g., fish heads, innards) as a fertiliser is less prevalent in the archaeological record, although a range of historical sources suggest its use throughout the medieval and post‐medieval periods.^6^, ^7^, ^16^, ^17^, ^18^, ^19^, ^20^ Fish waste, alongside other refuse, has also been identified as a component of anthrosols, many of which developed around rural settlements and urban centres with the expansion in fish consumption during the medieval period.^6^, ^21^, ^22^, ^23^

Fish appears to have been a particularly important fertiliser in North America,^24^ being used by Native Americans and European settlers in areas such as the Plymouth Colony.^25^ The same was true in nineteenth century New England, where a reported 27,000–37,800 lbs (13.5–18.9 tons) of fish were applied to an acre under cultivation in Marshfield, Massachusetts.^26^ Direct evidence for the use of fish as fertiliser has been identified in a preserved Native American field at Cape Cod.^27^ Modern studies have demonstrated the benefits of amendment with fish‐based compost to various plants, an application with commercial potential, and processed fish remains are an organic alternative to chemical fertilisers.^28^ In comparison, whilst there is a long history of using marine bird guano in South America, this form of fertiliser did not become widely used until the nineteenth and twentieth centuries in the Old World.^29^, ^30^ Taken together, it is evident that the benefits of marine resources as soil amendments have long been recognised.

Isotopic analysis of archaeobotanical material has been shown to be a powerful method for investigating past agricultural practices and land‐use patterns.^31^, ^32^, ^33^, ^34^, ^35^, ^36^, ^37^ Field experiments have indicated that the use of animal manure from terrestrial herbivores increases nitrogen isotope ratios (δ^15^N values) by up to 10‰ in cereals^35^, ^38^, ^39^ and 3‰ in legumes.^40^, ^41^, ^42^ In legumes, however, very intensive manuring is required to affect δ^15^N values, since legumes are nitrogen‐fixing through the use of symbiotic bacteria in root nodules: therefore, they typically exhibit plant δ^15^N values near to that of nitrogen in air (0‰).^43^, ^44^, ^45^

On the other hand, carbon isotope ratios (δ^13^C values) are thought to be minimally affected by manuring,^46^ with crop δ^13^C values primarily interpreted on crop water management practices^47^ or cultivation in different fields/soils.^31^, ^34^ However, other environmental factors (e.g., salinity, light intensity, temperature and nitrogen availability) can also affect plant δ^13^C values.^48^, ^49^ The relationship between plant δ^13^C values and manuring is not clearly understood and/or demonstrated, with both ^13^C enrichment and depletion being shown in experimental studies.^30^, ^35^, ^46^, ^50^

Plants uptake sulphur from the soil, normally in the form of sulphate (SO_4_^2−^), as well as from the atmosphere (SO_2_). Sulphur isotope analysis of archaeobotanical remains can be undertaken as part of a multi‐isotope approach together with δ^13^C and δ^15^N, and has the potential to provide information on the use of different soils/areas for cultivation as well as crop management practices.^51^ The application of sulphur isotope ratios (δ^34^S values) in the investigation of plant metabolism and environmental effects has received minimal attention,^52^, ^53^, ^54^ although there have been numerous reports on the uptake and metabolism of elemental sulphur.^55^, ^56^, ^57^, ^58^ Although there have been many modern crop studies using seaweed extracts or elemental sulphur, these have primarily focused on the growth response and uptake of sulphur and nitrogen in the plant,^30^, ^55^, ^59^, ^60^, ^61^ rather than tracing the effect of biofertilisation on sulphur isotopes. Szpak et al^62^ showed that the use of marine bird guano with very elevated δ^15^N values (e.g., > +20‰) had a significant effect on the δ^15^N values of maize (*Zea mays*), common bean (*Phaseolus vulgaris*) and summer squash (*Cucurbita pepo*) in a growth chamber experiment; a similar shift was also reported in δ^34^S values. They also reported no change in δ^34^S values in a northern Peru field experiment due to previous, long‐term application of ammonium sulphate. More recently, Blanz et al^63^ conducted a field experiment in Orkney, Scotland, to look at the effect of macroalgae fertilisation on hulled barley (*Hordeum vulgare*). They reported an insignificant change in barley grain δ^15^N values, but this is undoubtedly due to the small isotopic difference between the macroalgae (~ +6.7‰ ± 0.3‰) and a predicted field soil δ^15^N value; as no soil value was reported, we estimate the soil to be between +3.3‰ to +5.8‰ based on barley husk, grain and straw results.

Therefore, there is a need to investigate marine biofertilisation and isotopic uptake in crops grown in controlled outdoor experimental settings. In this study, we grew Celtic bean in an outdoor plot experiment using two different marine biofertilisation resources, macroalgae (i.e., seaweed) and Atlantic cod, over two growing seasons at the Botanic Garden, Durham University. The application of the biofertilisation followed methods which were identified in a review of relevant historical sources (outlined above). We hypothesised that the Celtic bean plant would change from background isotopic ratios (i.e., control) and incorporate the marine signature of the biofertilisers. The Celtic bean was naturally grown with no human intervention in 2017, but in 2018 we employed horticultural methods (e.g., watering, weeding) to determine if this had an effect on the stable isotope ratios. The plants were harvested ~16 weeks after sowing, measured and dried for subsequent whole tissue carbon, nitrogen and sulphur isotope analysis.

## 2 MATERIALS AND METHODS

### 2.1 Experimental design

The overall research aim of experimental archaeology is to aid interpretation of the material remains from the human past.^64^ This general approach does not seek to precisely observe, quantify and model experimental data, such as the data produced from modern agronomic research, but rather allows the researcher to gain new interpretive insights from the experimental process. Within the established theory, method and practice of experimental archaeology, we chose to conduct simulation experiments.^65^ These were based upon our working assumption that pulses (e.g., Celtic bean) were routinely grown using a form of ‘garden cultivation’ in small amended plots, based on archaeological evidence from across European history and prehistory.^66^

**FIGURE 1:**
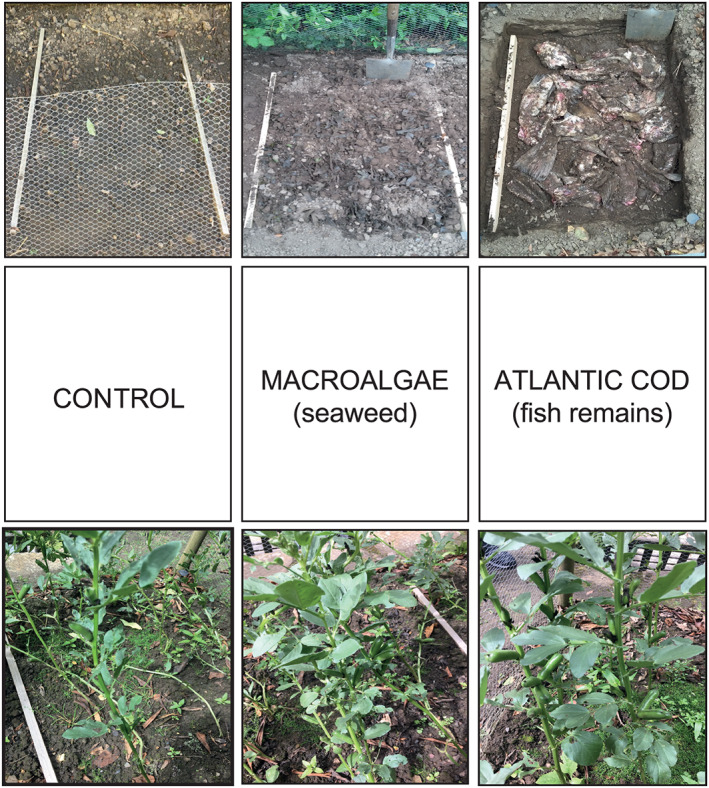
Experimental field plot set‐up with photos of the soil amendment and Celtic bean plants prior to harvesting. Note: The macroalgae was mixed in with the topsoil, whereas the Atlantic cod was buried at depth and not mixed [Color figure can be viewed at wileyonlinelibrary.com]

Celtic bean (*Vicia faba* L.) was cultivated in three 1 × 0.5 m outdoor plots at the Botanic Garden, Durham University, in the summer of 2017 and 2018 (Figure 1). The plots were surrounded with wire netting to prevent rabbits from entering and disturbing the plots. The variety selected for cultivation was Celtic Black broad bean, a heritage landrace of broad bean. This variety produces small, rounded seeds which are comparable with *Vicia faba* var. *minor* and morphologically similar to prehistoric and later finds of *V. faba*.^67^, ^68^ Twenty beans were planted directly in each plot during April of each year after soil amendment with the biofertilisers. All beans were sowed individually and spaced accordingly to avoid over‐crowding. The experimental area used in the Botanic Garden has not been previously used for any other research (e.g., no fertilisation or liming). One plot acted as a control (Plot 1c), and the other two plots were amended with naturally harvested biofertilisers: macroalgae (Plot 2m) and filleted Atlantic cod (Plot 3ac). The same individual plot areas were used in 2017 and 2018. Macroalgae (seaweed), samples, *Fucus vesiculosus* L. (bladder wrack), were collected from Staithes harbour, North Yorkshire, UK (54°33´N 00°47´W), less than a week prior to planting. Macroalgae from this site have previously been analysed for δ^15^N values.^69^ Living macroalgae were collected randomly from the foreshore and primarily consisted of non‐fertile tips of algae (~10 kg of undried material), although fertile tips may have also been present in the bulk sample. Within 24 h of collection the macroalgae were thoroughly mixed/mulched into the top 15 cm of soil in Plot 2m. This equates to ~100 t/ha and therefore, extremely intensive manuring/amendment.

North Sea Atlantic cod (*Gadus morhua*) were caught from *OUR LASS III* (Lockers Trawlers, http://www.lockerstrawlers.co.uk) using sustainable fishing management strategies and sold to Hodgson Fish (https://hodgsonfish.co.uk). The cod remains were provided filleted without freezing and immediately introduced into Plot 3ac without heads (~10 kg of fresh material): it is advised here at this stage that one should not transport the fish remains in your own vehicle! This also equates to ~100 t/ha and, therefore, extremely intensive manuring/amendment. In order to prevent vermin (i.e., rats) disturbing the plot, the top 15 cm of soil was removed from the plot and a compact layer of cod remains was placed at this depth. The removed soil was then replaced on top of the cod remains without mixing.

All Celtic bean plants were harvested when the majority of beans were mature. A variety of measurements were taken for each plant to get an estimate of plant biomass (i.e., productivity): number of plants in each plot, plant height, number of pods on each plant, pod length, number of beans in each pod, and bean dimensions (width, length, depth). All plant samples were partially air‐dried and then placed in a drying oven at ~40°C for a minimum of 48 h. Ten single‐entity beans were then randomly sub‐sampled from the total bean population for each plot, using a true random number generator: https://www.random.org.^70^ Five pods were then chosen randomly from the population of the pods previously sub‐sampled for ten beans: for example, for Plot 1c in 2017, seven pods were randomly sub‐sampled to produce the ten bean samples and the seven pods were then in turn sub‐sampled to generate the five pods. Five samples of stems and leaves were then taken from the five plants matching the pods. By doing this, the isotope measurements from the randomly sub‐sampled beans could be compared with their corresponding plant parts.

At the end of the experiment in each year, four random soil samples were taken from each plot. The top 2 cm of surface soil was removed, and the next 5 cm of soil sampled. The soil sample was placed in a drying oven at ~40°C for 48 h. The soil was lightly compressed and passed through a 1 mm stainless steel sieve: the <1 mm size fraction was ground into a fine powder and analysed for bulk stable isotope values.

### 2.2 Stable isotope analysis

Carbon and nitrogen isotope analyses of the experimental samples were performed using a ECS 4010 elemental analyser (Costech, Valencia, CA, USA) connected to a Delta V Advantage isotope ratio mass spectrometer (Thermo Scientific, Bremen, Germany) in the Stable Isotope Biogeochemistry Laboratory (SIBL) at Durham University. Isotopic accuracy was monitored through repeated analyses of in‐house standards (e.g., glutamic acid, δ^13^C = −11‰, δ^15^N = −7.5‰; IVA urea, δ^13^C = −43.26‰, δ^15^N = −0.56‰; col‐pure collagen, δ^13^C = −17.9‰, δ^15^N = 6.6‰; and spar calcite, δ^13^C = 2.9‰), before, during and at the end of the analytical sequence. All analyses included a series of international standards (e.g., USGS40, USGS24, IAEA‐600, IAEA‐CH‐3, IAEA‐N‐1, IAEA‐N‐2) at the start and end of the analytical sequence. These analytical standards provide an analytical range of −44‰ to 2.9‰ in δ^13^C, and −4.5‰ to 20.4‰ in δ^15^N. The analytical uncertainty in δ^13^C and δ^15^N was <0.1‰ (2 sd) for replicate analyses of the international standards and <0.2‰ (2 sd) for in‐house standards and replicate sample analysis. Total organic carbon and nitrogen data were obtained as part of the isotopic analysis using the in‐house standard glutamic acid (carbon = 40.82%, nitrogen = 9.52%).

Sulphur isotope analysis of the samples was performed in SIBL using an ECS 4010 elemental analyser connected to a Delta V Plus isotope ratio mass spectrometer. Isotopic accuracy was monitored through repeated analyses of in‐house standards (e.g., sulphanilamide) and international standards (e.g., IAEA‐SO‐5, IAEA‐SO‐6, NBS‐127). These analytical standards provided an isotopic range from −31‰ to 20.3‰ in δ^34^S. The analytical uncertainty in δ^34^S for replicate analyses of the international standards was <0.2‰ (2 sd) and <0.3‰ (2 sd) for in‐house standards and replicate sample analysis. Total sulphur data were obtained as part of the isotopic analysis using sulphanilamide (sulphur = 18.62%).

The sample weights varied depending on the material type in order to obtain an SO_2_ intensity of >800 mV for mass 64. Bulk leaf and bean tissue required >9 mg of sample, whereas bulk stem and pod material required >30 mg to achieve this mV criterion. Vanadium pentoxide (V_2_O_5_) is often used as an oxidant additive when performing sulphur isotope analysis; however, we have tested a range of V_2_O_5_ suppliers and noticed an appreciable sulphur blank when weighing more than 5 mg of V_2_O_5_ (Gröcke, unpublished data). Therefore, in SIBL we have opted to use tungstic oxide (WO_3_) as an additive: it has fewer health and safety issues and no measurable sulphur up to 100 mg (Gröcke, unpublished data). The “macro” oxygen setting on the Costech elemental analyser was used for all sulphur isotope analyses (standards and samples).

## 3 RESULTS

### 3.1 Celtic bean biomass

The biomass analysis of the Celtic bean plants is summarised in Table 1 (all raw data are provided in the supporting information). Where appropriate, a two‐tailed Student's t‐test was used to assess statistical significance between the amended (e.g., Plot 2m, Plot 3ac) and the control plants. Over the two experimental years (2017–2018), a total of 84 plants were grown and harvested, representing an overall germination success rate of 70%. The germination success rate was reduced in the amended plots in the order of 15% to 60%. The height of the Celtic bean plants ranged from 12 cm to 113 cm, with an average of 63 cm. There was no statistical difference in the height of the plants between the control and the amended plots.

Due to variability in plant germination, it is difficult to assess overall plot productivity. The number of pods produced in each plot varied by year and showed no systematic difference between control and amended. In 2017 there was a statistical significance in the difference in length of the pods between the control and amended plots, with the latter being significantly larger (Table 1). This difference was not observed in 2018.

**TABLE 1:** **Celtic bean biomass data for the control and experimental plots (c = control, m = macroalgae, ac = Atlantic cod) for years 2017 and 2018.** A two‐tailed student t‐test was calculated to assess statistical significance. Key: *p < 0.05; **p < 0.01; ***p < 0.001. Sd = standard deviation

	Plant			Pods			Beans						
	Number	Height (cm)	Sd	Number	Length (cm)	Sd	Number	Length, x (mm)	Sd	Width, y (mm)	Sd	Depth, z (mm)	Sd
**2017**													
Plot 1c	16	58	17	65	37.09	7.31	184	9.19	2.21	6.75	1.68	7.73	1.90
Plot 2 m	14	61	20	64	39.53*	7.86	186	9.93 **	2.37	7.07 *	1.84	8.30 **	2.07
Plot 3 ac	12	66	24	95	40.81**	7.56	250	10.26 ***	1.93	7.23 ***	1.43	8.59 ***	1.77
**2018**													
Plot 1c	20	69	18	82	42.40	7.85	274	10.46	1.87	10.80	1.70	10.84	1.67
Plot 2 m	8	71	12	52	42.56	6.29	159	10.80 *	1.70	7.72 ***	0.97	9.30 ***	1.31
Plot 3 ac	14	58	17	50	40.91	7.28	160	10.84 *	1.67	7.91 ***	1.13	9.60 ***	1.42
**TOTAL**													
Plot 1c	36	64	18	147	40.05	8.04	458	9.95	2.11	7.09	1.37	8.42	1.68
Plot 2 m	22	65	18	116	40.89	7.33	345	10.33 **	2.13	7.37 **	1.53	8.76 **	1.83
Plot 3 ac	26	61	20	145	40.84	7.44	410	10.49 ***	1.85	7.50 ***	1.36	8.99 ***	1.71

In each experimental plot there was no clear difference in the number of beans produced between the control and the amended plots in 2017 or 2018. However, the beans per plant ratio for each plot indicates that the amended plants produced more beans per plant (control = 13, macroalgae = 16, Atlantic cod = 16). The dimension of the beans was statistically larger in the amended plots than in the control plot (see Table 1), with the Atlantic cod plot showing the greatest size in beans. Each plant, from each plot in 2017 and 2018, was checked for Rhizobium symbiosis (i.e., counting of root nodules): we counted/observed no difference in our experimental plots and study.

### 3.2 Celtic bean stable isotopes

**FIGURE 2:**
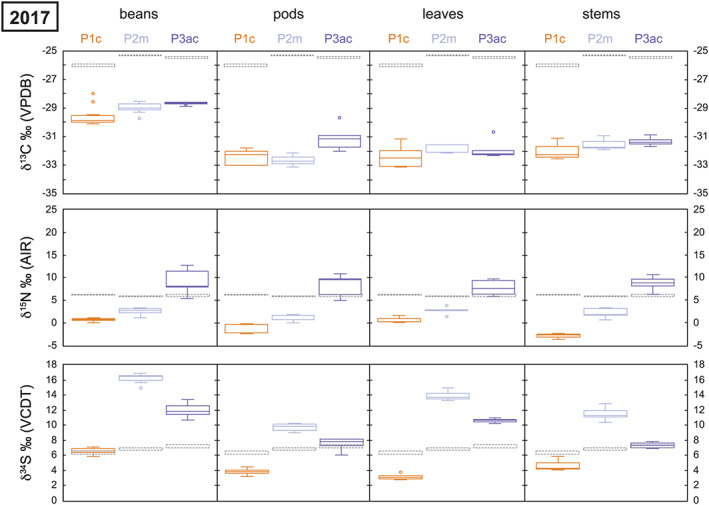
Box and whisker plot of the stable isotope data for the experimental plots in 2017. c = control, m = macroalgae, ac = Atlantic cod. Each graph column represents different components of the Celtic bean plants that were analysed (see Table 2 for the average results). Soil box and whisker results (dashed lines) for each plot are repeated in each column [Color figure can be viewed at wileyonlinelibrary.com]

**FIGURE 3:**
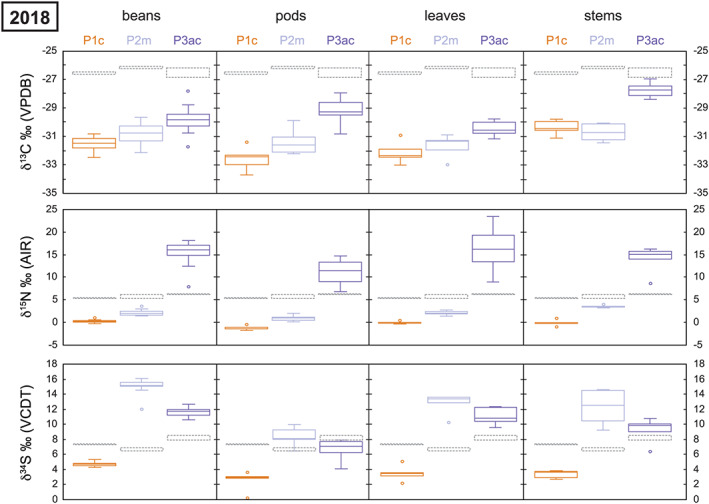
Box and whisker plot of the stable isotope data for the experimental plots in 2018. See Figure 2 for descriptions [Color figure can be viewed at wileyonlinelibrary.com]

A summary of the average Celtic bean plant tissue stable isotope ratios from 2017 and 2018 is presented in Table 2. All individual isotopic data is available in the supporting information. All stable isotope data are presented in Figure 2 (2017) and Figure 3 (2018).

The soil δ^13^C value for each plot in 2017 is fairly homogenous at between −25.5‰ and −26‰, whereas in 2018 all soils are more ^13^C‐depleted (−26‰ to −27‰): this depletion was also recorded in the control soil plot. The average δ^13^C value of the bladder wrack ranged from −14‰ in 2017 to −15.9‰ in 2018, whereas the value for the Atlantic cod averaged −14.1‰ in 2017 and −15.1‰ in 2018 (Table 3). Both biofertilisers are ^13^C‐enriched compared with the background soil of each plot. The beans all show a significant reduction in their δ^13^C value from 2017 to 2018: ^13^C‐depletion. All other plant tissues, however, show no depletion or enrichment in ^13^C between 2017 and 2018 in the control plot (Table 2; Figures 2 and 3). However, the two biofertilisation plots in 2018 record a significant ^13^C‐enrichment in beans, pods, leaves and stems compared with the control.

**TABLE 2:** Celtic bean stable isotope data for the control and experimental plots (c = control, m = macroalgae, ac = Atlantic cod). Sd = standard deviation

2017	Material	δ^13^C ‰ (VPDB)	Sd	δ^15^N ‰ (AIR)	Sd	δ^34^S ‰ (VCDT)	Sd	C:N atomic	Sd
Plot 1c	Bean (n = 10)	−29.6	0.7	0.8	0.3	6.6	0.4	8.0	1.0
	Pod (n = 5)	−32.5	0.5	−1.0	0.9	3.9	0.4	22.6	6.3
	Leaf (n = 5)	−32.4	0.7	0.8	0.6	3.3	0.3	9.0	0.1
	Stem (n = 5)	−32.0	0.6	−2.7	0.5	4.7	0.7	116.8	22.4
	Soil (n = 4)	−26.0	0.2	6.3	0.4	6.5	0.3	23.7	1.0
Plot 2 m	Bean (n = 10)	−29.0	0.3	2.7	0.7	16.2	0.6	8.3	0.6
	Pod (n = 5)	−32.7	0.4	1.1	0.7	9.7	0.5	32.7	7.3
	Leaf (n = 5)	−31.9	0.3	2.7	0.8	14.0	0.6	8.8	0.4
	Stem (n = 5)	−31.5	0.4	2.2	1.0	11.5	0.9	95.7	8.1
	Soil (n = 4)	−25.3	0.1	6.0	0.1	6.9	0.2	22.1	0.6
Plot 3 ac	Bean (n = 10)	−28.6	0.2	9.2	2.2	12.0	0.9	8.5	0.8
	Pod (n = 5)	−31.1	0.8	8.4	2.3	7.5	0.8	14.1	2.1
	Leaf (n = 5)	−31.9	0.6	7.8	1.5	10.7	0.3	8.6	1.1
	Stem (n = 5)	−31.4	0.3	8.8	1.4	7.4	0.3	94.7	14.5
	Soil (n = 4)	−25.5	0.1	6.4	0.7	7.3	0.3	23.9	1.4

2018	Material	δ^13^C ‰ (VPDB)	Sd	δ^15^N ‰ (AIR)	Sd	δ^34^S ‰ (VCDT)	Sd	C:N atomic	Sd
Plot 1c	Bean (n = 10)	−31.5	0.5	0.3	0.3	4.8	0.3	9.1	0.6
	Pod (n = 5)	−32.6	0.8	−1.2	0.4	2.6	1.2	36.7	3.1
	Leaf (n = 5)	−32.1	0.7	−0.1	0.3	3.5	0.9	12.6	1.3
	Stem (n = 5)	−30.4	0.5	0.0	0.6	3.6	0.5	58.3	14.2
	Soil (n = 4)	−26.5	0.1	5.3	0.1	7.3	0.3	24.6	1.4
Plot 2 m	Bean (n = 10)	−30.8	0.7	2.3	0.7	15.0	1.1	9.0	0.4
	Pod (n = 5)	−31.4	0.8	1.0	0.6	8.4	1.2	31.6	5.6
	Leaf (n = 5)	−31.7	0.7	2.1	0.5	12.7	1.3	12.8	1.8
	Stem (n = 5)	−30.7	0.6	3.5	0.3	12.4	2.1	72.9	5.2
	Soil (n = 4)	−26.2	0.2	5.8	0.5	6.7	0.3	27.3	2.2
Plot 3 ac	Bean (n = 10)	−29.8	1.0	15.2	2.9	11.6	0.6	8.3	0.7
	Pod (n = 5)	−29.2	1.0	11.1	2.9	6.6	1.4	37.8	2.1
	Leaf (n = 5)	−30.4	0.5	16.2	4.9	11.1	1.0	11.8	0.7
	Stem (n = 5)	−27.7	0.5	14.0	2.8	9.4	1.5	35.0	8.8
	Soil (n = 4)	−26.6	0.6	6.3	0.3	8.1	0.5	23.7	1.5

The soil δ^15^N value for each plot is fairly homogeneous at about 6‰ in 2017 and only slightly lower (more ^15^N‐depleted) in 2018. Therefore, it would appear that horticultural practices (e.g., weeding and watering) had no impact on the background soil δ^15^N values. However, this is not the case for the Celtic bean components which become significantly more ^15^N‐enriched in the macroalgae (Plot 2m) and Atlantic cod (Plot 3ac) experimental plots (see Figures 2 and 3). The amount of ^15^N‐enrichment in beans using the macroalgae biofertiliser (Plot 2m) is ~2‰ in 2017 and 2017, whereas for the Atlantic cod (Plot 3ac) it is ~ 8.5‰ in 2017 and up to ~15‰ in 2018 (Table 2). This significant increase in the δ^15^N value is caused by the biofertilisers (e.g., macroalgae and Atlantic cod) being ^15^N‐enriched compared with the background soil: the bulk tissue bladder wrack δ^15^N average ranged from 7.6‰ in 2017 to 8‰ in 2018, whereas that of the Atlantic cod muscle tissue averaged 15.4‰ in 2017 and 15.7‰ in 2018 (Table 3). Even the addition of natural biofertilisers in 2017 and 2018 had no significant effect on the bulk soil δ^15^N. Both biofertilisers are ^15^N‐enriched compared with the background soil of each plot.

**TABLE 3:** Biofertiliser stable isotope data for experimental plots (c = control, m = macroalgae, ac = Atlantic cod). Sd = standard deviation. * = only 2 samples

Common name	Species name	Material	Year	δ^13^C ‰ (VPDB)		δ^15^N ‰ (AIR)		δ^34^S ‰ (VCDT)	
				Average	Sd	Average	Sd	Average	Sd
Atlantic cod	*Gadus morhua*	Muscle	2017	−14.1	0.2	15.4	0.1	15.1	0.2
Atlantic cod	*Gadus morhua*	Muscle	2018	−15.1	0.5	15.7	0.2	15.0	0.2
Bladder wrack	*Fucus vesiculosus*	Fertile tip	2017	−14.0	1.4	7.8	0.3	18.3	0.04 *
Bladder wrack	*Fucus vesiculosus*	Fertile tip	2018	−15.4	0.7	7.6	0.4	18.3	–
Bladder wrack	*Fucus vesiculosus*	Non‐fertile tip	2017	−15.9	1.9	8.0	0.5	18.0	–
Bladder wrack	*Fucus vesiculosus*	Non‐fertile tip	2018	−15.4	1.3	7.7	0.5	18.2	0.07 *

The soil δ^34^S value was more variable between the plots in 2017 and 2018 than the δ^13^C and δ^15^N values (Table 2). The control soil δ^34^S value (Plot 1c) was 6.5‰ in 2017 and 7.3‰ in 2018, whereas that of the macroalgae plot (Plot 2m) was 6.9‰ and 6.7‰, respectively. The Atlantic cod soil δ^34^S value (Plot 3ac) significantly increased from 7.3‰ in 2017 to 8.2‰ in 2018 (Figures 2 and 3). Therefore, it is unclear if horticultural practices (e.g., weeding and watering) had an impact on background soil δ^34^S values. Unlike for δ^13^C and δ^15^N, these marine biofertilisers had the greatest effect on δ^34^S in the Celtic bean plants. The bean δ^34^S values in Plot 1c averaged 6.6‰ in 2017 and 4.8‰ in 2018, whereas in Plot 2m they averaged 16.2‰ in 2017 and 15‰ in 2018, and in Plot 3ac they averaged 12‰ in 2017 and 11.6‰ in 2018. The bulk tissue bladder wrack δ^34^S average ranged from 18.3‰ in 2017 to 18‰ in 2018, whereas Atlantic cod muscle tissue averaged 15.2‰ in 2017 and 15‰ in 2018 (Table 3). Both biofertilisers are ^34^S‐enriched compared with the background soil of each plot.

## 4 DISCUSSION

### 4.1 Marine biofertilisation effects on growth

There was variability in germination success rates between plots, with a higher success rate from the control plot than from the amended plots. We suggest that this is a function of the increased ability of the amended soils to contain pests, such as slugs and snails, due to increased organic components and the concomitant increase in nitrogen in the amended soils. We hypothesise that this may have been the case in the past and would have been an important consideration when undertaking significant and sustained amendment of soils for crop cultivation. In addition, there was no statistical difference in the plant heights between the control and the amended plots. Marine biofertilisation does not seem to increase the amount of green vegetation (e.g., leaves, stems), and this may have been a factor in choosing crops in the past when considering secondary fodder products from the crop. However, the bean/plant ratio was higher in the amended plots than in the control crop, indicating the potential for greater bean productivity in amended soils.

Statistically, the bean size increased in both amended crops compared with the control crop, with Plot 3ac having the greatest increase. Therefore, this may mean that larger Celtic beans recovered from archaeological sites may in fact have been cultivated in amended soils. Combining these basic archaeobotanical metrics (see supporting information) with stable isotope ratios is a very promising avenue for future research into understanding crop management practices, although the effect of carbonisation on bean shape, bean size and stable isotope ratios will need to be carefully considered at each site.^51^, ^68^

### 4.2 Carbon isotope discrimination in Celtic beans

Each plant component (e.g., stem, leaf, pod and seed) has a different purpose and therefore has biochemical reactions that would naturally differentiate the uptake of ^13^C *versus* ^12^C (*idem*, nitrogen). The average δ^13^C values of each component are summarised in Table 2 and graphically presented in Figures 2 and 3. Each plant component is ^13^C‐depleted compared with the soil for all plots, irrespective of the type of biofertilisation used: the only plant tissues that are very close are stems in Plot 3ac (Figure 3). The beans are more ^13^C‐enriched than other plant tissues in all the experimental plots in 2017. Beans are predominantly made up of carbohydrates (>60%) which are ^13^C‐enriched compared with other plant components such as cellulose, lignin and lipids.^71^ However, this relationship breaks down in 2018 when the plots were managed.

The δ^13^C value of plants is predominantly controlled by the isotopic composition of CO_2_ and photosynthetic pathway (C_3_, C_4_, CAM).^71^ Since carbon within plant tissues ultimately derives from photosynthesis,^71^ it was predicted that the different biofertilisers added to Plot 2 (macroalgae) and Plot 3 (Atlantic cod) would have little effect on the δ^13^C value. Although the bean δ^13^C values shifted accordingly towards the values of the ^13^C‐enriched biofertilisers, this shift was far more enhanced during 2018 than during 2017 (see Figures 2 and 3). Another environmental factor that has significant control on the plant δ^13^C value is water availability during growth.^47^ The average bean δ^13^C values for each plot became lower (Plot 1c = 1.9‰, Plot 2m = 1.8‰, Plot 3ac = 1.2‰) from 2017 to 2018. The most likely explanation for this shift in bean δ^13^C is the increased water availability in 2018 when the crop was watered regularly: lower watering regimes (i.e., water stress) are known to cause plants to become more ^13^C‐enriched due to stomatal conductance.^46^, ^48^, ^71^, ^72^ The relationship between δ^13^C and water availability in temperate environments (as opposed to semi‐arid environments) where water is not a limiting factor on plant growth has been little studied and the relationship is not clear. Changes in the δ^13^C value could reflect a number of factors: (1) soil composition (i.e., increased organic matter increasing water retention); (2) plant biomass/dry matter production; and (3) rates of N_2_‐fixation and those of photosynthesis (since the two are very closely linked).^73^ However, all the other plant tissue components analysed in this study actually show the reverse relationship: less negative δ^13^C values in 2018 than in 2017. The exact mechanism causing this relationship is not obvious, but it may be due to increased uptake of soil ^13^C in Plot 2m and Plot 3ac over 2017 to 2018. Previous studies have indicated that plants can take up to 5% carbon from the active soil reservoir.^74^ In addition, changes in the biosynthetic pathways for each plant component as a response to marine biofertilisation may also play a part.

**FIGURE 4:**
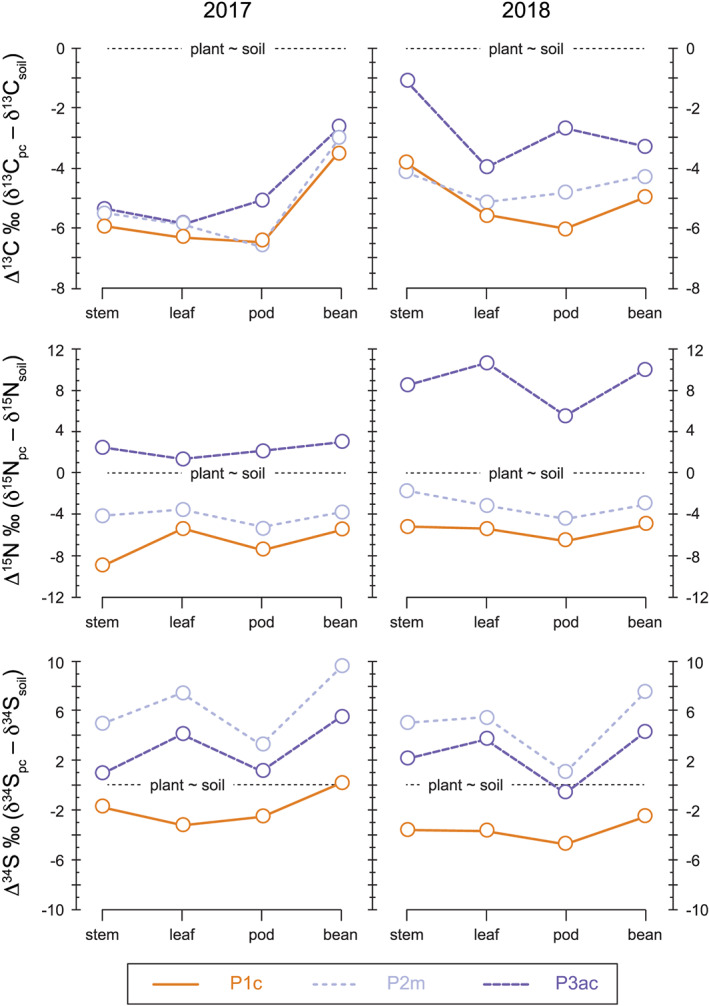
Δ values for carbon, nitrogen and sulphur isotope ratios from each of the plant components (see text for description). A positive Δ value means that there is enrichment of ^13^C, ^15^N and ^34^S, respectively, in that system compared with soil (background) values. pc = plant component. See Figure 2 for descriptions [Color figure can be viewed at wileyonlinelibrary.com]

In order to investigate the effect of biofertiliser sources on the uptake and incorporation of specific isotopes a discrimination factor (Δ = δ_a_ – δ_b_) can be used. In the case of this study, the soil isotope ratio from the control experiment (Plot 1c) can be used as a background signature since Plot 2m and Plot 3ac have been amended. Therefore, Δ^13^C = δ^13^C_pc_ – δ^13^C_soil_, where pc is the specific plant component (e.g., bean, pod, leaf, stem). The Δ^13^C values for the specific plant tissues are illustrated in Figure 4. The discrimination factor is within error (0.5‰ ± 2 sd) for stem–soil, leaf–soil and bean–soil in 2017. The lack of similarity in the 2017 pod–soil discrimination may actually be the result of not homogenising the pod tissue sufficiently (Figure 4).

During 2017 no crop management practices were employed and Δ^13^C remains fairly consistent between each plant component and soil. However, in 2018 crop management was employed on all three plots (e.g., watered and weeded). This change in crop management had a significant effect on Δ^13^C (Figure 4). Although Plot 1c and Plot 2m recorded similar values in all components to that of the soil (with a minor difference in pod–soil discrimination), Plot 3ac was significantly different and recorded much less carbon isotope discrimination between plant and soil (Figure 4). The significant difference in the Plot 3ac Δ^13^C values may also be caused by the more extreme ^13^C‐enriched value of the Atlantic cod (~ −14.5‰) compared with the Staithes macroalgae (~ −15.5‰).

**FIGURE 5:**
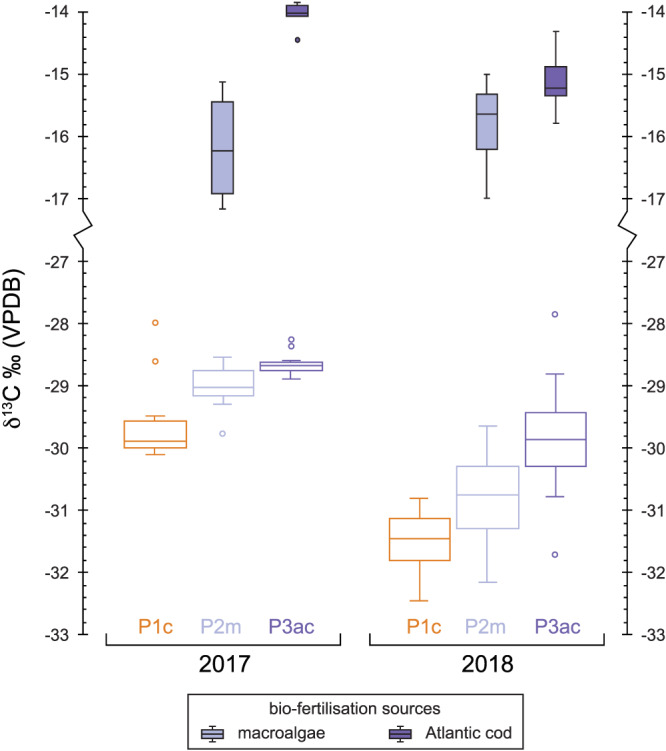
Carbon isotope ratio box and whisker plot of the beans from experimental plots in 2017 and 2018, compared with the δ^13^C value of the biofertilisers used. Note the change in the y‐axis scale [Color figure can be viewed at wileyonlinelibrary.com]

An illustrative plot of only the bean δ^13^C results is presented in Figure 5 to show the distinct differences in δ^13^C for each experimental plot and biofertilisation sources, and the changes between 2017 and 2018. The effect of crop management practices between 2017 and 2018 can clearly be seen in Figure 5 where less watering caused δ^13^C to be less negative in 2017 than in 2018 when the plots were well‐watered and managed. The effect on bean and plant δ^13^C values of adding a ^13^C‐enriched biofertiliser is not fully determined in this study, as the experiment would require the same management practices to be repeated from one year to the next. Despite this, our results demonstrate that biofertilisation with marine resources has the potential to have an effect on δ^13^C values.

### 4.3 Nitrogen isotope discrimination in Celtic beans

It has previously been shown that the nitrogen‐fixing beans can produce higher δ^15^N values under significant soil amendment (e.g., manure)^40^, ^42^; this has also been recorded in amino acid δ^15^N values.^41^ Only extremely intensive (i.e., >80–100 t/ha) manuring caused ^15^N‐enrichment (up to 3‰), probably due to a reduction in the proportion of nitrogen obtained via N_2_‐fixation in favour of nitrogen uptake from the ^15^N‐enriched soil.^42^ In the case of this study, the macroalgae applied to soils had a similar δ^15^N value to manure used in a previous experiment by Treasure et al.^42^ However, the Atlantic cod δ^15^N values are much higher (Atlantic cod muscle tissue = ~15.5‰) (see Table 3).

As opposed to the carbon isotope ratios of each plant component, the nitrogen isotope ratios are significantly different between each plot in this study (see Figures 2 and 3). Nitrogen accumulation and assimilation in legumes are ultimately driven by a combination of plant biomass (and by extension photosynthesis) and soil N availability.^75^, ^76^ The biofertilisers increased the δ^15^N values of all components of the Celtic bean plant relative to the control plot plants. This enrichment in ^15^N is more prominent in 2018 (Figure 3), especially in Plot 3ac. This significant increase is hypothesised to be the result of plants assimilating ^15^N‐enriched nitrogen from the soil. Where legumes are assimilating soil nitrogen, the plant δ^15^N value is expected to increase with the fertiliser δ^15^N value: animal manure has a comparatively low δ^15^N value (e.g., cattle manure <5‰),^5^ whereas seaweed (7.8‰) and, in particular, fish have high δ^15^N values (15.5‰). Others have also recorded high δ^15^N values for fish fertilisers, potentially >16‰.^77^, ^78^, ^79^ An example of the relationship between plant and fertiliser δ^15^N values is provided by Szpak et al,^5^ who observed increases of 19‰ in the values for common bean fertilised with seabird guano, reflecting the high δ^15^N values of seabird guano (>20‰).

A nitrogen isotope discrimination plot can be generated by calculating the difference between the plant components and the control soil δ^15^N values (Δ^15^N = δ^15^N_pc_ – δ^15^N_soil_) (Figure 4). The Δ^15^N for all tissues in the control plot gave values > − 4‰ in both years, indicating that N_2_‐fixation was the dominant process in these Celtic bean plants, considering that the background soil δ^15^N value is around 5‰ (see Figure 3). Nitrogen discrimination was less for Plot 2m, but the δ^15^N value of this plot never reached that of the soil (e.g., Δ^15^N = 0‰) (Figure 4). In comparison, Plot 3ac had a positive Δ^15^N indicating that the main source of nitrogen in these plants was from the Atlantic cod muscle tissue (>6 wt % N). The Δ^15^N for Plot 3ac was much higher in 2018 as a result of phosphorus release from the soils^73^ and/or increased water use efficiency. Increased water use efficiency is known to improve soil nitrogen uptake,^76^ which potentially caused more uptake of ^15^N in 2018. In addition, in the presence of sulphur, nitrogen uptake has been shown to increase in crops.^80^, ^81^, ^82^ Therefore, by using a biofertiliser with high sulphur content (e.g., macroalgae > 2 wt % S, marine fish > 1 wt % S), nitrogen uptake efficiency would increase. In addition, uptake of nitrogen from the soil, as opposed to atmospheric N_2_, will yield a reduction in N_2_‐fixation rates. High soil nitrogen availability has been widely demonstrated to suppress N_2_‐fixation rates in legumes.^75^

**FIGURE 6:**
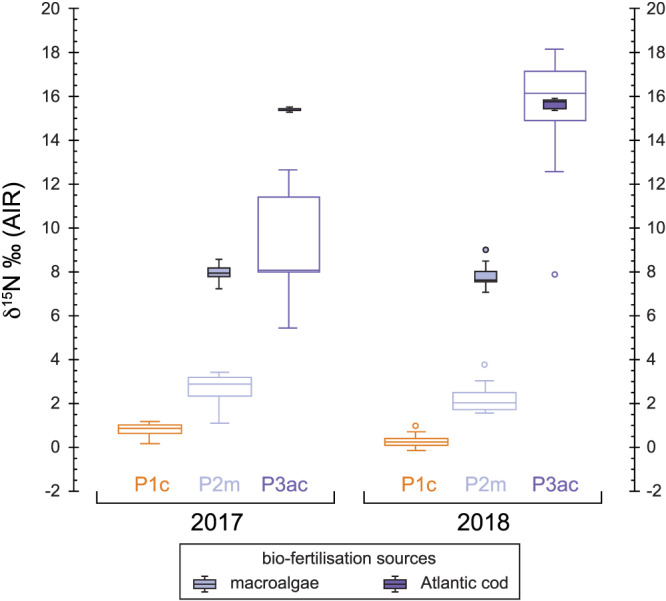
Nitrogen isotope ratio box and whisker plot of the beans from experimental plots in 2017 and 2018, compared with the δ^15^N value of the biofertilisers used [Color figure can be viewed at wileyonlinelibrary.com]

The δ^15^N values of the Celtic beans in Plot 3ac ranged from 8‰ up to 18‰, with an average of 15.2‰: very similar to the Atlantic cod, ~15.5‰ (Figure 6). This degree of scatter may in fact relate to the randomness of the cod remains at 15 cm, and therefore changes in the presence or amount of muscle tissue (e.g., proteins/nitrogen) available to the root system (see Figure 6). Why some bean δ^15^N values exceed the average value for Atlantic cod muscle tissue is uncertain. Further breakdown of the biofertilisers during the winter of 2017–2018 may have taken place, combined with utilisation of ^14^N from other flora/fauna: therefore, giving a soil that is more ^15^N‐enriched than in the previous year.

### 4.4 Sulphur isotope discrimination in Celtic beans

Sulphur plays an important role in the legume‐rhizobia system of nitrogen‐fixation in plants.^81^ The addition of marine biofertilisers with elevated sulphur content to the experiment had significant effects on nitrogen assimilation. In fact, sulphur uptake in the Celtic beans probably mimicked that of nitrogen in that it was efficiently absorbed and transferred into the plant. The beans showed the greatest isotopic shift of the order of 6‰ to 10‰ compared with beans from Plot 1c (Figures 2 and 3). In fact, these plants have shifted from a terrestrial δ^34^S signal (Plot 1c) to a marine value in just one growing season.

**FIGURE 7:**
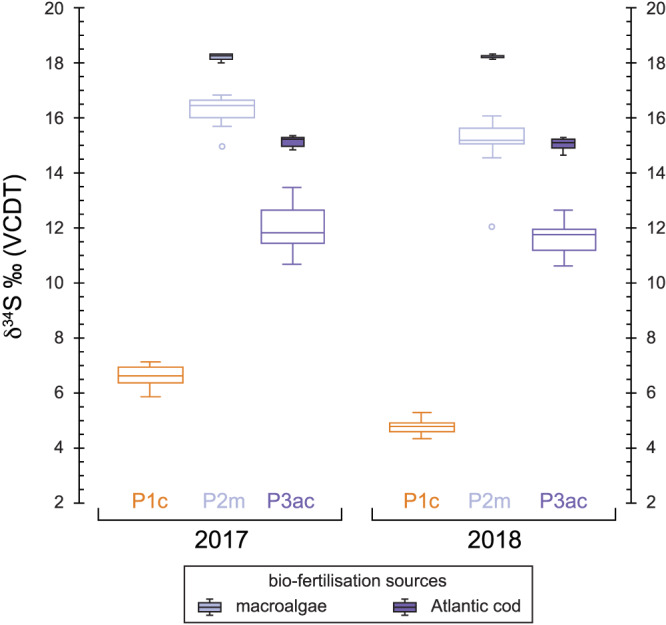
Sulphur isotope ratio box and whisker plot of the beans from experimental plots in 2017 and 2018, compared with the δ^34^S value of the biofertilisers used [Color figure can be viewed at wileyonlinelibrary.com]

Sulphur isotope fractionation, Δ^34^S (Δ^34^S = δ^34^S_pc_ – δ^34^S_soil_), can be employed to assess the degree of change from a background soil to the biofertiliser source (Figure 4). There is a clear shift towards greater fractionation in Plot 2m and Plot 3ac. It is greatest in Plot 2m as the δ^34^S value of the macroalgae (~18‰) is more positive than that of Atlantic cod muscle tissue (~15‰) (Figure 4). Due to its importance in forming essential amino acids (cysteine and methionine),^83^ it is likely that the sulphur would have been rapidly incorporated into the plant and deposited in the protein‐rich beans. Figure 7 indicates this, as the beans very rapidly shifted towards the δ^34^S value of the introduced biofertilisers. Plot 1c recorded a decline in Δ^34^S, suggesting incorporation of ^32^S into the plant tissues and subsequent enrichment of the soil ^34^S reservoir, hence causing the 2018 soil to have a higher δ^34^S value than soil sampled at the end of 2017. Compared with the other discrimination plots for carbon and nitrogen there is a consistent pattern in the plant component Δ^34^S values in Plot 2m and Plot 3ac (Figure 4). The cause of this is currently not known but may be due to the spatial distribution and deposition of amino acids in the plant.

### 4.5 Archaeological implications of marine biofertilisation

The application of marine biofertilisers on Celtic bean crops has been shown to have a significant effect on the biomass and isotopic composition of all plant tissues, especially the beans. Whilst animal manure was the most widely used fertiliser in the past, a range of other biofertilisers were also widely applied.^7^ In particular, marine biofertilisers, such as seaweed and fish, are likely to have been particularly important in areas near to coastlines, especially throughout the Medieval period.^6^, ^7^ In fact, marine biofertilisation may have had significant impact inland, since macroalgae have been recovered from archaeological sites up to 50 km from the coast in northern England.^84^ At the least, our results demonstrate the impact of this on Celtic bean, one of the most common legume crops,^67^, ^68^ although our results have wider implications for other crops, such as cereals. The levels of biofertiliser applied here can be characterised as intensive, and therefore would be restricted in the scale of cultivation, probably in small plots in close proximity to settlements.^85^ It should be noted here that the use of freshwater biofertilisers (e.g., freshwater fish, animal remains and macrophytes) may also lead to elevated δ^15^N and δ^34^S values.

Taking this into account, the δ^15^N and δ^34^S shifts reported in this study may have many implications when interpreting isotopic signatures of archaeological humans, flora and fauna after the arrival of agriculture. For example, even where cereals are cultivated on intensively manured soils, grain δ^15^N values rarely exceed >10‰.^32^ Similarly, high bean δ^15^N values (i.e., >3%) are unlikely in crops cultivated with herbivorous animal manures, even where applications are extremely intensive.^40^, ^42^ Recent archaeological investigations have reported anomalous crop (e.g., cereals) δ^15^N values (>10‰) in the Neolithic to Medieval periods of Europe.^86^, ^87^ These anomalous δ^15^N values have typically been disregarded in the discussion, although in light of this study they may be reinterpreted to indicate biofertilisation using fish remains (e.g., Plot 3ac, Figure 6); of course, this will depend on how scattered/dense the fish remains are in the crop soil.

**FIGURE 8:**
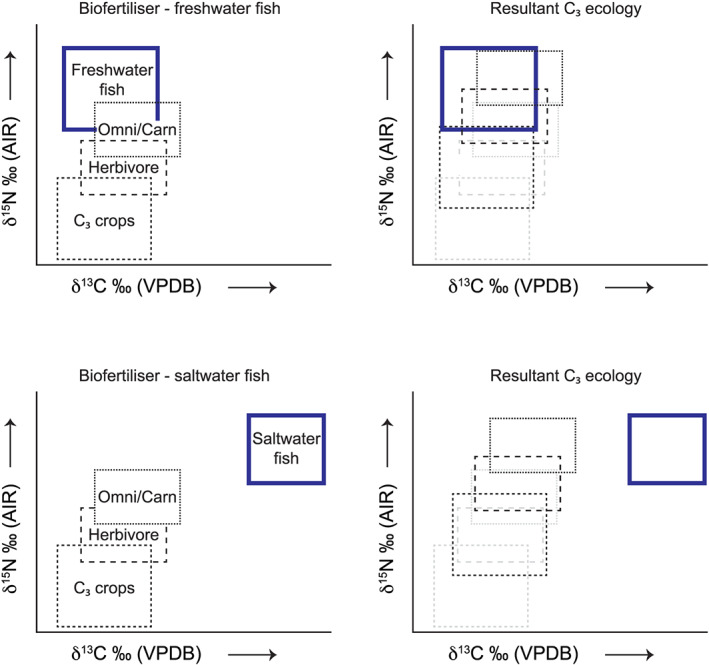
Idealised C_3_ δ^13^C *versus* δ^15^N plots for an ecosystem (left), and resultant plots if there were amendment strategies (right) using freshwater fish (top right) and saltwater fish (bottom right). See text for discussion [Color figure can be viewed at wileyonlinelibrary.com]

**FIGURE 9:**
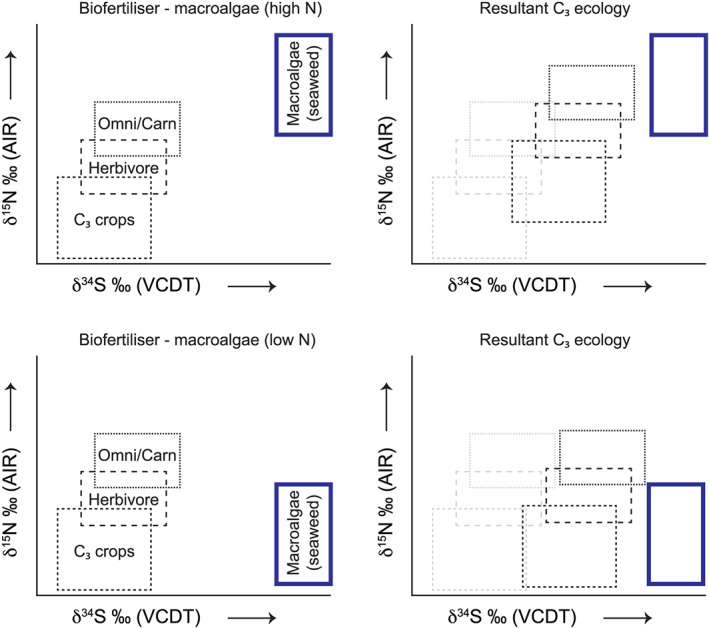
Idealised C_3_ δ^34^S *versus* δ^15^N plots for an ecosystem (left), and resultant plots if there were amendment strategies (right) using more positive δ^15^N macroalgae (top), and less positive δ^15^N macroalgae (middle). See text for discussion [Color figure can be viewed at wileyonlinelibrary.com]

**FIGURE 10:**
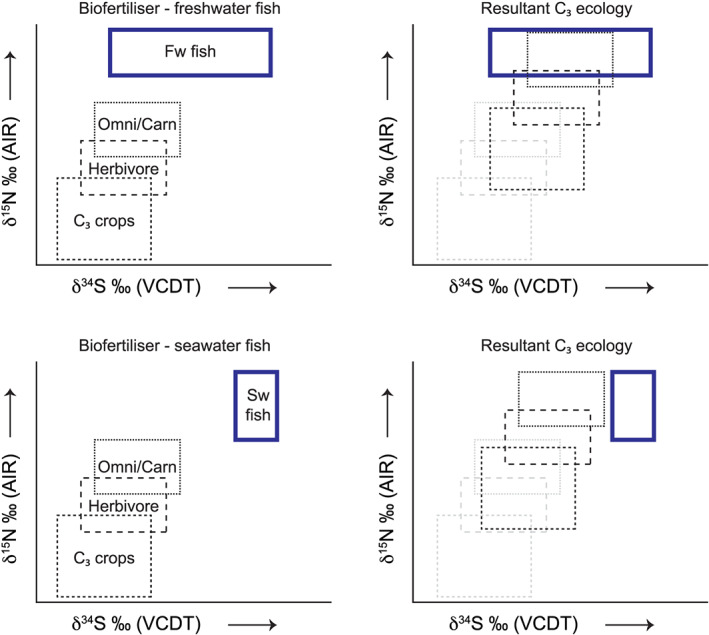
Idealised C_3_ δ^34^S *versus* δ^15^N plots for an ecosystem (left), and resultant plots if there were amendment strategies (right) using freshwater fish (top), and saltwater fish (bottom). See text for discussion [Color figure can be viewed at wileyonlinelibrary.com]

A method to distinguish marine from freshwater biofertilisation would be difficult using just δ^13^C and δ^15^N values (see sections 4.2 and 4.3), but in the case of δ^34^S it is more feasible, although this may not be possible when the sample from freshwater fish is enriched in ^34^S (e.g., seawater sulphate) due to bacterial sulphate reduction.^88^, ^89^ Although archaeological communities living by the ocean may not rely on marine resources for dietary consumption, they may potentially use seaweed for biofertilisation. In this scenario, the human population may record elevated δ^15^N (see Figure 6) and δ^34^S values (see Figure 7) that would suggest the presence of marine resources in their diet, but the δ^13^C values may still be quite low (see Figure 5). Idealised stable isotope plots for δ^13^C *versus* δ^15^N are depicted in Figure 8. Whether freshwater or seawater fish were used as a biofertiliser it would be difficult to differentiate them just using δ^13^C and δ^15^N, but either way they increase the δ^15^N value by at least a trophic level. However, when combining δ^34^S with δ^15^N the effect of marine biofertilisation (e.g., macroalgae, fish) is more apparent (Figures 9 and 10). It should be noted here that macroalgae δ^15^N values are affected by nitrogen loading in the region and are species‐specific (Gröcke, unpublished data); therefore, the use of a specific δ^15^N value for macroalgae is not applicable.

As an example of how interpretations can vary as a result of biofertilisation, Nehlich et al^90^ interpreted a shift in δ^34^S from ~4‰ to ~14‰ in archaeological human collagen as indicative of a shift in freshwater fish. However, in this scenario, freshwater fish had generally low δ^15^N values (~7‰) compared with humans (ranging from 11.5‰ to 15‰)^90^: typically, outside a trophic level shift of ~3–5‰.^91^ An alternative interpretation, based on this study, could be that freshwater fish were predominantly being used as a crop biofertiliser, hence elevating the δ^15^N value by more than one trophic level (see Figure 10). Consumption of the biofertilised crop would incorporate the δ^34^S freshwater signal and a higher δ^15^N value. Therefore, by not including agricultural amendment strategies this may lead to an inaccurate reconstruction of human palaeodiet using fruits.^92^ Potentially, there are other studies that will require reconsideration when including the impact of crop biofertilisation.

Another aspect that needs consideration is that of sea spray. The direct effect of sea spray on plant δ^34^S values has not been adequately demonstrated in a modern setting directly from plants. Living near to the coast does not always equate to plants having elevated δ^34^S values, as it relies on: (1) predominant wind direction during the growing season; (2) salt tolerance of the plants; and (3) rate of sea spray deposition from the coast. Therefore, it is incorrect to assume that if a community was living near the coast the area was affected by sea spray. Plant δ^34^S data from coastal environments in the north east of England suggest that there is little to no enrichment in ^34^S indicative of sea spray (Gröcke 2019, unpublished data). In addition, plants and fish living near/in estuaries affected by freshwater sulphate and anaerobic processes will typically exhibit lower δ^34^S values than seawater sulphate.^93^ Hence, the interpretation of δ^34^S as a recorder of proximity to the coast in archaeological material (e.g., human collagen) is not simple. Further research is urgently required on the interplay of soil, water, plant and animal δ^34^S values around coastal, estuarine and lake environments.

Many archaeological studies predominantly focus on human/animals or plants, but rarely a combination of both. Based on this study and others,^5^, ^32^, ^35^, ^42^, ^62^, ^63^, ^87^ it is important to also assess the impact of biofertilisation on the stable isotope ratios of archaeological fauna. This study has further highlighted other factors that need to be considered when interpreting ancient diets, such as:

1. Animal foddering: many domesticated animals are fed on a variety of agricultural crop by‐products.^94^ This study has shown that all components of the plant are enriched during biofertilisation and not just the human‐consumed part of the plant.
2. There is very strong evidence for fish fertilisation in North America^95^; therefore, it may be worth considering its impact on ancient farming practices and palaeodiets in this region of the world.
3. During the Medieval period there was increased reliance on the use of marine resources in different areas of the world, such as north‐west Europe^96^ and the North Atlantic region.^97^ Therefore, biofertilisers such as seaweed and fish (as products of domestic rubbish) may have led to a general increase in δ^15^N and δ^34^S value around agricultural or urban settlements compared with more inland communities. However, transportation of coastal resources inland may have been very likely during this time period.
4. It is evident from this study that further research is required on other biofertilisers (cesspits, industrial waste, marl, sea sand, etc.) and not just animal manures.^5^ In addition, different sources of manure (e.g., human, sheep, pigs) are likely to cause isotopic variability and future experimentation will be required to assess the extent of this.

## 5 CONCLUSIONS

An experimental study was performed on Celtic bean crops grown in marine‐amended soils over two consecutive years, and subsequently analysed for carbon, nitrogen and sulphur isotope ratios. The soils were amended with marine biofertilisers, macroalgae and Atlantic cod. This experiment was performed to determine if the marine isotopic signatures of these fertilisers were incorporated into the biomass of the bean plants. The marine biofertilisers had only a minimal effect on the biomass calculations of the bean plants; however, the numbers of beans per plants were significantly greater in the amended plots. Conversely, the nitrogen and sulphur isotope signatures of the marine‐amended beans were ^15^N‐ and ^34^S‐enriched compared with the control plot; this was more significant in the second year of the experiment. The δ^15^N values of the beans in the Atlantic cod plot were elevated by more than one trophic level. The marine δ^34^S signature of the biofertilisers was rapidly incorporated into the plant tissues. This study highlights the impact on the interpretation of δ^15^N and δ^34^S from archaeological humans and animals that were consuming plants/crops grown with marine biofertilisers. Human movement of marine products inland would complicate matters even more, by not being limited to a marine isotope interpretation based solely on proximity to the coast. Additional modern experimental research using other biofertilisers is required.

6

### PEER REVIEW

The peer review history for this article is available at https://publons.com/publon/10.1002/rcm.8985.

## Supporting information


**Table S1** Supporting Information

**Table S2** Supporting Information
